# The Effects of a Powdered Meal Replacement on Inflammation, Gut Microbiota, and Metabolism in People With Excess Body Weight—A Randomized Controlled Trial

**DOI:** 10.1016/j.tjnut.2026.101595

**Published:** 2026-05-15

**Authors:** Julia Montenegro, Camila LP Oliveira, Nguyen Khoi Nguyen, Anissa M Armet, Aloys Berg, Arya M Sharma, Laurie Mereu, Cristiane Cominetti, Sunita Ghosh, Patrice D Cani, Caroline Richard, Jens Walter, Carla M Prado

**Affiliations:** 1 Department of Agricultural, Food and Nutritional Science, University of Alberta, Edmonton, AB, Canada; 2 Institute of Parasitology, Faculty of Agricultural and Environmental Sciences, McGill University, Montreal, QC, Canada; 3 Department of Medicine, Faculty of Medicine, University of Freiburg, Freiburg, Germany; 4 Department of Medicine, University of Alberta, Edmonton, AB, Canada; 5 Faculty of Nutrition, Federal University of Goiás, Goiânia, Brazil; 6 Department of Medical Oncology, University of Alberta, Edmonton, AB, Canada; 7 Department of Public Health Sciences, Henry Ford Hospital, Detroit, MI, United States; 8 Metabolism and Nutrition research group (MNUT), Louvain Drug Research Institute (LDRI), UCLouvain, Université Catholique de Louvain, Brussels, Belgium; 9 Walloon Excellence in Life Sciences and BIOtechnology (WELBIO), WELBIO Department, WEL Research Institute, Wavre, Belgium; 10 Institute of Experimental and Clinical Research (IREC), Université catholique de Louvain, Brussels, Belgium; 11 APC Microbiome Ireland, Biosciences Building, University College Cork, Ireland; 12 Department of Medicine, University College Cork, National University of Ireland, Cork, Ireland

**Keywords:** obesity, meal replacement, inflammation, gut microbiome, body composition

## Abstract

**Background:**

Excess body weight is associated with chronic low-grade inflammation and metabolic abnormalities, such as insulin resistance, and dyslipidemia.

**Objectives:**

This study aimed to assess the impact of a soy protein-yogurt-honey powdered meal replacement (PMR) on inflammation, gut microbiota, and metabolism in individuals with excess body weight and in weight-stable conditions.

**Methods:**

The Powdered meal REplacement on MetabolIsm and gUt Microbiota (PREMIUM) Study was a 12-week, parallel-arm, randomized controlled trial. Participants (body mass index: 25–37 kg/m^2^) were randomized into either control (CON; usual diet, *n* = 34) or PMR (2 daily doses added to usual diet, *n* = 29) groups, maintaining a stable body weight. Assessments occurred at baseline, week 6, and week 12, and included inflammation markers (primary outcome: IL-6), gut microbiota diversity and composition (secondary outcome), metabolic blood markers (glucose and lipid profile), body composition (via dual-energy X-ray absorptiometry), and dietary intake. Data of completers were analyzed by 2-way repeated measures analysis of variance or generalized estimating equations with Bonferroni-corrected post hoc tests. Between-group differences in changes over time are expressed as mean and 95% confidence intervals.

**Results:**

Adherence to PMR was 98% of total doses, which increased protein intake [6.53% (5.04%, 8.02%), *P* < 0.001] and decreased fat intake [−5.23% (−7.10%, −3.35%), P < 0.001] compared with CON. By design, body weight remained stable. There were no changes in IL-6 [−0.01 (−0.47, 0.45) pg/mL, *P* = 0.412], with a low statistical power of 13.7%. Minor changes in gut microbiota composition included an increase in relative abundance of *Subdoligranulum* (0.72 Log_2_ fold-change, *q* = 0.002). In exploratory outcomes, PMR increased lean soft tissue [0.57 (0.12, 1.02) kg, *P* = 0.014] and reduced total cholesterol [−0.33 (−0.58, −0.08) mmol/L, *P* = 0.01] and low-density lipoprotein cholesterol [−0.28 (−0.46, −0.10) mmol/L, *P* = 0.003].

**Conclusions:**

In this population, PMR intake did not improve chronic low-grade inflammation and had limited effects on gut microbiota, but was associated with improvements in lean soft tissue and lipid profile that warrant further exploration.

This trial was registered at clinicaltrials.gov as NCT03235804 on August 1, 2017.

## Introduction

Excess body weight (i.e., BMI ≥25 kg/m^2^ indicating overweight and obesity) and, more specifically, excess adiposity are associated with elevated concentrations of inflammatory markers, including IL-6, IL-8, TNF-α, and C-reactive protein (CRP) [1], a condition known as chronic low-grade systemic inflammation. This inflammatory state contributes to various metabolic abnormalities [2], such as insulin resistance (i.e., impaired insulin function leading to elevated fasting insulin and glucose) and dyslipidemia (increased triglycerides, total cholesterol, and LDL cholesterol, and reduced HDL cholesterol) [3]. Additionally, hormones modulating metabolism (nutrient and energy metabolism) and appetite are often disrupted, including leptin resistance [4], downregulation of adiponectin [5], diminished postprandial suppression of ghrelin, and lower production of glucagon-like peptide-1 (GLP-1), and peptide tyrosine-tyrosine (PYY) [4].

Individuals with excess body weight also exhibit an altered composition of the gut microbiota (i.e., the community of microorganisms colonizing the intestinal tract) compared with healthy normal-weight controls [6,7]. This may alter the functional capacity of the microbiota, contributing to metabolic abnormalities. For example, increased gut permeability observed in obesity allows higher absorption of bacterial products, triggering a proinflammatory response, further exacerbating chronic low-grade systemic inflammation and metabolic dysfunction [8,9]. A healthy gut microbiota performs several functions potentially disrupted in obesity, such as supporting gut barrier integrity [10], competing against pathogenic and opportunistic bacteria for nutrients and adhesion sites [11], and stimulating appetite-suppressing gut hormones (e.g., GLP-1 and PYY) [12], among other functions [13]. Therefore, gut microbiota represents a potential target for antiobesity interventions.

Various dietary approaches have been developed for weight loss, including meal replacements [[14], [15], [16], [17]]. Among these, a soy protein-yogurt-honey powdered meal replacement (PMR) has demonstrated improvements in weight loss [[18], [19], [20], [21], [22], [23], [24], [25], [26], [27], [28]], glucose control (reduced fasting glucose and insulin resistance markers) [18,[20], [21], [22], [23], [24], [25], [26], [27]], lipid profiles (decreased triglycerides, total cholesterol, LDL cholesterol, and increased HDL cholesterol) [25,26], hormonal regulation (lowered fasting leptin and postprandial ghrelin) [18,19,22,28], and inflammation markers (decreased CRP and IL-6) [27]. However, it remains unclear whether the observed metabolic improvements occur indirectly through weight loss or whether PMR directly improves inflammation and metabolism. Moreover, only one study has investigated PMR effects on inflammation [27], and its impact on gut microbiota and the extent to which gut microbiota modulation may mediate health improvements have not been examined. The components of PMR may affect inflammation markers and gut microbiota composition. Soy protein contains bioactive peptides, polyphenols like isoflavones and phenolic compounds, and other bioactive compounds. Soy intake has been shown to reduce CRP [29], improve lipid profile and glycemic control [30]. Yogurt contains bioactive peptides and conjugated linoleic acid, potentially inducing health benefits such as reduced CRP and increased gut microbiota α-diversity [31,32]. Yogurt also contains live bacteria with potential probiotic effects, but PMR is unprobeable to contain live microbes due to its processing [33]. Honey has phenolic compounds that could decrease inflammation [34] and oligosaccharides that promote enrichment of beneficial bacteria and inhibit pathogenic ones in animal models [35].

Considering the potential unexplored effects of PMR and its components, this study aimed to evaluate whether PMR intake can improve inflammation and metabolism in individuals with excess body weight under conditions of weight stability, and if such effects are linked to changes in gut microbiota composition. We hypothesized that PMR intake would reduce proinflammatory markers, modulate gut microbiota composition, improve markers of glucose regulation and lipid profile, and improve energy expenditure and body composition even without weight loss.

## Methods

### Study design and participants

The impact of a Powdered meal REplacement on MetabolIsm and gUt Microbiota (PREMIUM) study was a 12-week, parallel-arm, randomized controlled trial (RCT), and its research protocol has been previously detailed [36]. Potential participants attended a screening visit, during which informed consent was obtained and eligibility assessed. Eligible participants were adults (aged 18–50 y) with excess body weight (BMI, 25–37 kg/m^2^), body fat percentage (BF%) >20% for males and >25% for females, without comorbidities, and with stable weight (<5 kg variation in the previous 6 mo). Individuals who were smokers, had chronic diseases or acute infections, pregnant or lactating, or taking medications that alter study outcomes (e.g., antibiotics or anti-inflammatory medications), taking protein supplements, prebiotics or probiotics, or performing >3 h/wk of vigorous physical activity were excluded.

Sex-stratified randomization was performed on Microsoft Office Excel using a predetermined list of computer-generated numbers and assigned by an investigator not involved in recruitment through randomly permuted blocks (2 and 4 sizes). The allocation list was concealed from the investigator responsible for participant assignment by hiding the cells. Although the investigator had technical access to the allocation list, it was not consulted during eligibility assessment or assignment, and participants were allocated sequentially according to the order of enrollment. Enrolled individuals (*n* = 88) were randomly assigned (1:1) to either the control (CON) group, maintaining their usual diet, or the PMR group, adding 2 servings (50 g each) of a soy protein-yogurt-honey formula (Almased Wellness-GmbH) mixed with 250 mL of water to their usual diet as morning and afternoon snacks. Nutritional information for the PMR is described in Supplementary Table 1. Because there was no equivalent inert placebo, treatment allocation could not be blinded. It was expected that adding PMR to the habitual diet would lead participants to naturally adjust their dietary intake due to its satiety-inducing effects. This would result in an overall better dietary pattern as their habitual diet likely reflects a North-American pattern. Therefore, group differences in dietary patterns were considered part of the intervention rather than confounding effects.

After a screening visit to determine eligibility, participants attended study visits at baseline, week 6, and week 12 for data collection. Initiation of medications potentially affecting study outcomes (e.g., antibiotics and corticosteroids) was a discontinuation criterion. Recruitment and data collection occurred between April 2019 and December 2023 at the University of Alberta, Edmonton, Canada. Procedures were approved by the University of Alberta Human Research Ethics Board and adhered to ethical standards.

### Data collection

Fasting blood samples were collected at each visit, and stool samples were collected within the same week using DNA/RNA Shield (Zymo Research). Body composition was evaluated by dual-energy X-ray absorptiometry (DXA; GE Lunar and enCORE software v13.60, General Electric Company) for the assessment of BF%, fat mass (FM; kilograms), lean soft tissue (LST; kilograms), and bone mineral content (kilograms). Energy metabolism was assessed via whole-room indirect calorimetry (WRIC) while fasting, and gas exchange in the WRIC was analyzed minute-by-minute by the Advance Optima AO2000 Series CO_2_ analyzer (ABB Automation GmbH) and the OXYMAT 6 O_2_ analyzer (Siemens AG), and data were analyzed using the PMCSS Software V.1.8 (Pennington Metabolic Chamber Software Suite, Pennington Biomedical Research Center). A mean of 30 min resting metabolic rate (kilocalories per minute) was multiplied by 1440 to determine resting energy expenditure (REE, kilocalories per day), and the volume of CO_2_ exhaled divided by the volume of O_2_ inhaled determined the respiratory exchange ratio (RER) [37]. Participants completed the Godin-Shephard leisure-time physical activity questionnaire [38,39] and were asked to maintain their usual physical activity levels.

Participants recorded their daily weight and were asked to maintain a stable body weight throughout the study (<3% change from baseline). If weight fluctuated by >2%, counseling from a registered dietitian was provided to facilitate a return to baseline weight. Those in the PMR group recorded each PMR dose and completed weekly appetite assessments using a visual analog scale at 5 time points: fasting upon waking, immediately before, and 30 min after each dose (morning and afternoon). Appetite sensations evaluated were hunger, satisfaction, fullness, and prospective food consumption (PFC) [40], with weekly AUC derived from these measurements. Dietary intake in both groups was evaluated using the Automated Self-Administered 24-h Recall (ASA24-Canada) [41], administered 3 times (2 weekdays, 1 weekend day) at weeks 1, 6, and 12, and once weekly during the remaining weeks. Dietary analyses in weeks 1, 6, and 12 were based on the average of 3 recall days in each week. Adverse events were assessed weekly via phone calls.

### Biological samples analysis

The primary and secondary outcomes of this study were changes in IL-6 concentrations and gut microbiota composition over time, respectively. IL-6 was selected as the primary outcome because, among the most studied cytokines, it is more closely related to chronic low-grade inflammation than CRP [42,43] and shows stronger associations with adiposity and chronic diseases than TNF-α [[44], [45], [46]]. Although CRP is widely used, it is a downstream acute-phase reactant that increases rapidly in response to infections or tissue injury, resulting in nonspecific high day-to-day variation due to transient and subclinical events [42]. In contrast, IL-6 acts upstream in the inflammatory cascade and more directly reflects metabolic and immunological pathways relevant to chronic low-grade inflammation [43]. Thus, IL-6 is a more reliable and mechanistically specific biomarker for detecting intervention-related changes in this study. Other blood markers and clinical parameters were exploratory outcomes.

Thyroid-stimulating hormone, glucose, triglycerides, total cholesterol, HDL cholesterol, LDL cholesterol, non-HDL cholesterol, and high-sensitivity CRP (hs-CRP) were measured in serum samples by DynaLIFE Medical Labs. Hs-CRP was measured on 2 consecutive days to eliminate the possibility of subclinical infection; for values below the detection limit (<0.5 mmol/L), a value of 0.2 mmol/L was assigned. Remaining blood markers were quantified on-site. All assays included blank and CON samples in addition to the reference standards. Lower and upper limits of quantification (LOQ) and intra-assay coefficient of variations (CVs) are presented. Free glycerol (LOQ = 16–488 μM, CV = 6.4%) and free fatty acids (LOQ = 110–3320 μM, CV = 5.5%) were measured in triplicates of serum samples using colorimetric assay kits (Zen-Bio Inc). The remaining blood markers were measured in duplicates of plasma samples using electrochemiluminescence immunoassays (Meso Scale Discovery). Specifically, a custom V-PLEX inflammatory panel was used to measure IL-6 (LOQ = 0.48–1402 pg/mL, CV = 9.1%), IL-8 (LOQ = 0.35–1169 pg/mL, CV = 5.8%), IL-10 (LOQ = 0.19–755 pg/mL, CV = 10.6%), and TNF-α (LOQ = 0.22–732 pg/mL, CV = 10.6%). A custom U-PLEX metabolic panel quantified insulin (LOQ = 0.35–125 mIU/L, CV = 4.0%), leptin (LOQ = 0.07–247 ηg/mL, CV = 7.4%), active ghrelin (LOQ = 2.4–4650 pg/mL, CV = 7.0%), active GLP-1 (LOQ = 0.01–32 pmol/L, CV = 5.1%), and total PYY (LOQ = 9–11489 pg/mL, CV = 4.1%) concentrations in plasma with protease inhibitors. The HOMA-IR was calculated using fasting glucose and insulin concentrations [47]. Adiponectin was measured using a singleplex assay (LOQ = 0.09–25 mg/mL, CV = 2.4%), and the leptin-to-adiponectin ratio (LAR) was calculated.

Stool samples were stored frozen at −80°C until study completion, then aliquoted (250 μL) and shipped to the University of Minnesota Genomic Center for analysis. DNA was extracted using DNeasy PowerSoil Pro HTP kits (Qiagen). Amplification targeted the V5–V6 regions of the 16S rRNA gene using the primer pair 784F [5′-RGGATTAGATACCC-3′] and 1064R [5′-CGACRRCCATGCANCACCT-3′]. Sequencing was performed on the MiSeq v3 platform (Illumina) with a 2 × 300 bp paired-end run. To minimize batch effects, all samples were sequenced in a single run.

### Data analysis

Sample size calculation was based on a previous RCT [48] that reported a decrease in IL-6 among healthy women with excess body weight consuming soy protein (the main component of the PMR; −6.76% ± 36.95%), and an increase in the group consuming a control protein (mix of casein, whey, and egg white; 17.62% ± 35.92%) over 12 mo without weight loss. The standardized effect size (ES) was calculated as the difference between-group means divided by the pooled standard deviation (24.38/36.44), resulting in Cohen’s *d* = 0.669. Sample size was calculated *a priori* using G∗Power (V.3.1.9.2) using a 2-tailed t-test for the difference between 2 independent groups without accounting for multiple comparison corrections. A total of *n* = 74 participants was required, considering a power of 80%, a significance of 5%, and an allocation ratio of 1:1. Considering a 20% attrition rate, a total of *n* = 88 participants (*n* = 44 per group) was targeted.

Missing data were imputed using the last observation carried forward method for body composition variables, and blood markers analyzed on-site outside LOQ were imputed using the Monte Carlo multiple-imputation method. The normality assumption was evaluated using the Shapiro–Wilk W-test and by inspecting histograms and Q-Q plots to evaluate normality of residuals, downstream analyses were selected based on data distribution as appropriate. Descriptive data are expressed as mean ± SD for normally distributed variables, or as median (interquartile interval) for non-normally distributed variables. Analysis was conducted on data from all completers to evaluate time, group, and time-by-group interaction effects, without correcting for adherence and protocol deviations. Normally distributed variables were analyzed using 2-way repeated measures analysis of variance (RM-ANOVA) and Bonferroni-adjusted post hoc tests. Prior to RM-ANOVA, Mauchly’s Test of Sphericity was performed, and, if violated, Greenhouse–Geisser correction was applied. Levene’s Test was also used to evaluate homogeneity of variance prior to RM-ANOVA. If RM-ANOVA assumptions were violated, generalized estimating equations (GEE) with an autoregressive (AR1) working correlation with a binomial distribution and logit function was performed. Model adequacy was evaluated by inspecting goodness-of-fit, working correlation matrix, Pearson residuals, and the covariance and correlation matrices of parameter estimates. Significant effects were followed up with post hoc Bonferroni-adjusted nonparametric tests (Mann–Whitney U-test or Wilcoxon signed-rank test) as a conservative approach given the small sample size. Significant post hoc between-group differences are described as mean (95% confidence interval), unless otherwise specified. To minimize false positive results, effects were corrected for multiple testing across biologically related outcomes (e.g., energy metabolism parameters, cholesterol fractions) using Benjamini–Hochberg false discovery rate (FDR). Changes in weekly appetite sensations in the PMR group were assessed with 1-way RM-ANOVA. Analyses of sex interactions were conducted by including sex as a covariate (males = 0, females = 1), and results with significant FDR-corrected effects are presented, except when only the sex effect was significant and expected (e.g., BF%, REE). Correlations were evaluated using Bonferroni-corrected Spearman’s rank correlation coefficients (rho) and classified as strong (≥±0.7), moderate (≥±0.4 and ≤±0.7), or weak (≤±0.4) [49]. ES were expressed as partial η^2^ and the corresponding Cohen’s *f* for RM-ANOVA, and as Wald’s χ^2^ with the derived Cramér’s *V* for GEE. The observed power (OP) was calculated based on Cohen’s *f* and Cramér’s *V*. ES and OP values are reported for significant outcomes except in sex-interaction analysis. Clinical data analyses were performed using IBM SPSS Statistics for Windows (v.29; International Business Machines Corporation) with statistical significance set at *P* < 0.05. Figures were generated using GraphPad Prism (v.10.3.0, GraphPad Software, LLC).

Gut microbiota data analysis was performed using R (v4.4.2, R Foundation for Statistical Computing), and bioinformatic procedures were performed on the QIIME 2 platform [50]. Multiple quality–control steps were applied to raw sequencing data using the DADA2 algorithm [51], including primer removal, sequence trimming, chimera removal, decontamination, and filtering (<3 counts/sample or present in <10% of samples). Samples containing <2000 reads were excluded from downstream analysis. Sequences were classified into Amplicon Sequence Variants (ASV) using the classify-sklearn algorithm [52] against Silva database (v.138) tailored for the primer set via the RESCRIPt pipeline [53]. Data were centered log-ratio transformed to account for compositionality. Metrics of α-diversity (Shannon index, Inverse Simpson index, and observed ASVs) and β-diversity (Bray–Curtis distance) were computed using the phyloseq [54] and microbiome [55] R packages. Sex was included as a covariate (males = 0, females = 1) in statistical models to adjust for potential confounding effects. Changes to α-diversity metrics were assessed using linear mixed models. β-diversity was visualized using Principal Coordinate Analysis (PCoA) ordination graphs based on Bray–Curtis distances. Comparisons of β-diversity PCoA were conducted with permutational multivariate analysis of variance (PERMANOVA), controlled for intraindividual variation to prevent inflation of significance due to the nonindependence of repeated-measure samples. Bray–Curtis distances were further compared using linear mixed models to assess within-individual shifts and between-individual differences. The Linear model for Differential Abundance (LinDA) analysis evaluated variations of taxa (phylum, genus, and ASV levels) relative abundance using the MicrobiomeStat R package and controlled for sex [56]. All linear models (analysis of α-diversity, β-diversity distances, and LinDA) included group, timepoints, and sex as fixed effects and Subject ID as a random effect to account for repeated measurement within individuals. A time-by-group interaction was not included because the primary objective was to estimate marginal effects of group and time while accounting for within-subject correlation. Including interaction terms across thousands of taxa would substantially increase the multiple-testing burden, reduce power for low-prevalence taxa, and produce unstable estimates in sparse abundance matrices. This additive specification provides interpretable marginal effects and preserves model stability. Potential interactions were subsequently probed in post hoc analyses using planned contrasts (estimated marginal means) for taxa showing significant main effects after FDR correction. FDR-adjusted *P* values (*q*) were considered statistically significant (*q* < 0.05) or indicative of trends (*q* < 0.1). Results are expressed as relative abundance unless otherwise specified.

## Results

### Baseline characteristics

**FIGURE 1:**
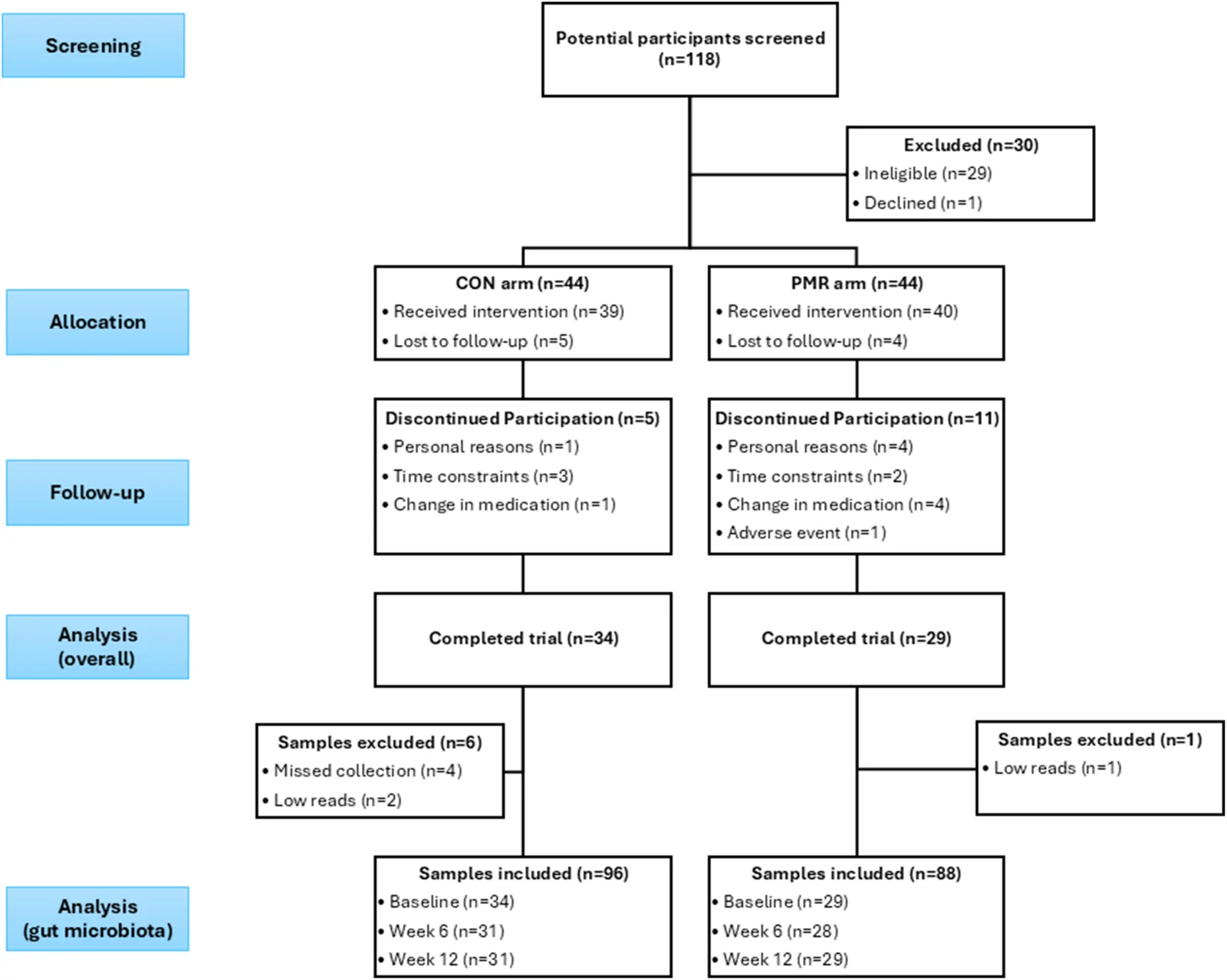
CONSORT flow diagram depicting progression of participants. CON, control group; PMR, powdered meal replacement group.

A total of *n* = 118 individuals attended the screening visit, *n* = 88 met the eligibility criteria and were randomly assigned to either the CON or PMR group (*n* = 44 per group). Before baseline visits, 5 CON and 4 PMR participants withdrew (lost to follow-up), leaving *n* = 39 in CON and *n* = 40 in PMR at baseline (Figure 1). After baseline, another 5 CON and 11 PMR withdrew or were removed. Most dropouts were attributed to personal reasons and time constraints (lacked time to complete study tasks and/or to attend visits). Only 1 withdrawal was due to an adverse event unrelated to the PMR intake. Additionally, 5 participants were removed after starting medications that were exclusion criteria according to the study protocol [36]. The analysis, therefore, included *n* = 63 completers (CON, *n* = 34, 77.3%; PMR, *n* = 29, 65.9%), and most were female (CON, *n* = 24, 70.6%; PMR, *n* = 18, 62.1%) (Figure 1). The higher dropout rate in PMR group could introduce bias and potentially skew the results.

For descriptive purposes, baseline comparisons between completers and dropouts (*n* = 12) are detailed in Supplementary Table 2. There were minimal differences when not considering group allocation, except for higher active GLP-1 in dropouts (*P* = 0.021). Within-group comparisons showed that CON dropouts (*n* = 3) had higher RER (*P* = 0.048), whereas PMR dropouts (*n* = 9) had higher BF% (*P* = 0.016) and leptin (*P* = 0.032) compared with completer counterparts. However, given the small number of dropouts, particularly within groups, these analyses were underpowered to detect small or moderate differences and should be interpreted cautiously. Differences between completers and dropouts could introduce attrition bias and potentially affect the interpretation of outcomes. Among completers, only LST in females differed between groups at baseline (*P* = 0.018), but not when adjusted for height^2^.

Most blood parameters were within normal ranges at baseline (Table 1), and a few had values outside reference ranges. Specifically, 3 had glucose concentration >5.6 mmol/L (1 in CON, 2 in PMR) [57]; 6 had fasting insulin ≥12 mIU/L (3 in each group); and 1 in PMR had HOMA-IR >4.65, indicating insulin resistance [58]. For lipid profile, 12 participants had triglycerides >1.7 mmol/L (5 in CON, 7 in PMR) [57,59]; 6 had total cholesterol >5.2 mmol/L (1 in CON, 5 in PMR) [59]; 7 had HDL cholesterol concentrations <1.0 mmol/L (3 in CON, 4 in PMR) [57, 59]; and 2 in PMR had LDL cholesterol >3.5 mmol/L [59]. For hs-CRP, 16 participants had concentrations >3 mg/L (11 in CON, 5 in PMR), indicating chronic low-grade inflammation, and of whom 5 exceeded 10 mg/L (4 in CON, 1 in PMR), indicating a high level of chronic inflammation or ongoing infection [60]. Those with hs-CRP >10 mg/L had similar values on consecutive days and throughout the study, suggesting a high level of chronic inflammation rather than infection.

**TABLE 1:** Baseline characteristics of participants in the control (CON) and powdered meal replacement (PMR) arms.

Variable		CON (*n* = 34)	PMR (*n* = 29)
Sex (females: males)		24:10	18:11
Age (y)		28 (23, 33)	28 (23, 35)
Height (m)		1.67 ± 0.09	1.71 ± 0.10
Weight (kg)		80.2 (72.4, 86.7)	83.2 (79.3, 88.7)
BMI (kg/m^2^)		28.1 (27.3, 29.8)	29.1 (26.6, 30.4)
Body fat (%)^1^	M	33.49 ± 4.07	32.57 ± 5.80
	F	40.96 ± 6.19	39.18 ± 4.86
Fat mass (kg)^1^	M	29.51 ± 5.88	29.69 ± 7.87
	F	32.12 ± 9.33	31.37 ± 4.45
LST (kg)^1^	M	55.05 ± 4.15	58.12 ± 9.94
	F	42.57 ± 4.03	46.01 ± 5.02
LST index (kg/m^2^)^1^	M	18.16 ± 0.67	18.60 ± 1.83
	F	15.90 ± 1.13	16.29 ± 1.11
REE (kcal/d)		1662 (1488, 1863)	1748 (1561, 1909)
RER		0.837 ± 0.038	0.843 ± 0.044
Triglycerides (mmol/L)		1.05 (0.74, 1.37)	1.23 (0.82, 1.64)
Free glycerol (μM)		56.5 (35.8, 76.0)	45.7 (32.0, 75.2)
Free fatty acids (μM)		600.1 (315.9, 771.5)	492.8 (306.6, 730.7)
Total cholesterol (mmol/L)		4.21 ± 0.69	4.52 ± 0.75
HDL cholesterol (mmol/L)		1.34 ± 0.29	1.35 ± 0.37
LDL cholesterol (mmol/L)		2.40 ± 0.61	2.62 ± 0.58
Non-HDL cholesterol (mmol/L)		2.87 ± 0.66	3.17 ± 0.66
Glucose (mmol/L)		4.9 (4.6, 5.1)	4.8 (4.6, 5.1)
Insulin (mIU/L)		5.94 (4.62, 7.21)	5.76 (4.67, 8.34)
HOMA-IR		1.29 (1.00, 1.68)	1.18 (0.98, 1.77)
IL-6 (pg/mL)		2.07 (1.41, 2.59)	2.00 (1.42, 3.13)
IL-8 (pg/mL)		3.35 (2.78, 4.18)	3.51 (2.88, 4.02)
IL-10 (pg/mL)		1.40 (1.11, 2.06)	1.34 (1.01, 1.79)
TNF-α (pg/mL)		2.56 (2.12, 3.99)	2.56 (1.80, 3.73)
hs-CRP (mg/L)		1.0 (0.2, 3.4)	0.6 (0.2, 2.2)
TSH (mIU/L)		1.83 (1.24, 2.29)	1.71 (1.16, 2.53)
Leptin (ηg/mL)		6.62 (4.13, 12.14)	6.10 (4.14, 8.70)
Adiponectin (mg/mL)		4.72 (3.67, 6.31)	4.62 (3.49, 6.85)
Active ghrelin (pg/mL)		240.2 (188.9, 366.8)	224.8 (184.3, 288.5)
Active GLP-1 (pmol/L)		1.05 (0.62, 1.48)	0.85 (0.62, 1.75)
PYY (pg/mL)		118.3 (96.5, 158.4)	141.4 (108.7, 194.5)

Data expressed as mean ± SD if normally distributed or as median (interquartile interval) if non-normally distributed.

Abbreviations: GLP-1, glucagon-like peptide-1; hs-CRP, high-sensitivity C-reactive protein; LST, lean soft tissue; PYY, peptide tyrosine-tyrosine; REE, resting energy expenditure; RER, respiratory exchange ratio; TSH, thyroid-stimulating hormone.

1 Body composition data expressed in each sex: M = males and F = females.

### Measures of adherence to the study protocol

**FIGURE 2:**
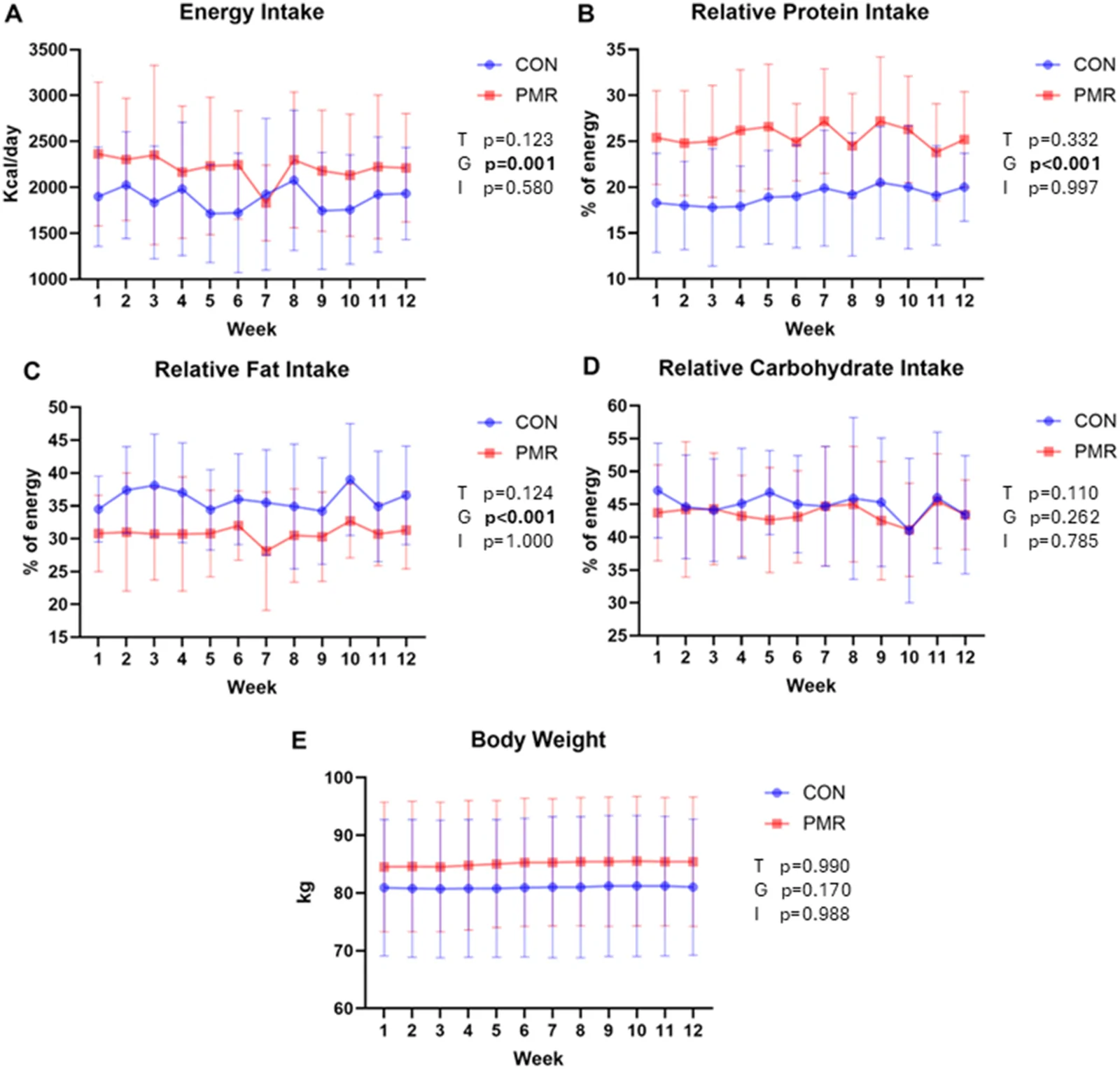
Weekly dietary intake and body weight evaluated in participants in control (CON, *n* = 34) and powdered meal replacement (PMR, *n* = 28) groups using RM-ANOVA. (A) Energy (kilocalories per day). (B) Protein (percentage of energy). (C) Fat (percentage of energy). (D) Carbohydrate (percentage of energy). (E) Body weight (kilograms). False discovery rate-corrected for multiple testing effects included: time (T), group (G), and time-by-group interaction (I). CON, control group; PMR, powdered meal replacement group.

Self-reported adherence to the PMR was high [median, 98% (96.4%, 100%)]. Figure 2A to D shows weekly energy intake and macronutrient distribution (percentage of total energy). There was a group effect on energy intake (*P* = 0.001), with PMR consuming more calories than CON [333.8 (138.9, 528.7) kcal/d]. Compared with CON, PMR had higher (*P* < 0.001) absolute protein intake [49.22 (38.08, 60.36) g/d) and relative protein intake [6.53% (5.04%, 8.02%)]. Relative fat intake was lower in PMR [−5.23% (−7.10%, −3.35%), *P* < 0.001], although absolute fat intake did not differ between groups [0.92 (−8.36, 10.19) g/d, *P* = 0.844]. PMR also had higher absolute carbohydrate intake [32.17 (7.81, 56.34) g/d, *P* = 0.017], whereas relative carbohydrate intake did not differ from CON [−1.30% (−3.60%, 1.00%); *P* = 0.262]. Additionally, there were group effects on sugar [22.97 (9.23, 36.72) g/d, *P* = 0.001] and dietary fiber [3.04 (0.46, 5.62) g/d, *P* = 0.017] intake, both were higher in PMR than in CON. No group differences were observed for dietary cholesterol, saturated fatty acids, monounsaturated fatty acids, and polyunsaturated fatty acids. Adding PMR to habitual diet resulted in a total mean protein intake of 1.71 ± 0.37 g/kg/d (males = 1.65 ± 0.35 g/kg/d; females = 1.74 ± 0.38 g/kg/d) and provided adequate amounts of all essential amino acids (Supplementary Table 3). Conversely, those in CON group had a lower (*P* < 0.001) protein intake of 1.05 ± 0.34 g/kg/d (males = 1.10 ± 0.30 g/kg/d, *P* = 0.002; females = 1.03 ± 0.36 g/kg/d, *P* < 0.001). Differences in dietary intake reflect the nutritional composition of PMR and the expected displacement of foods.

By design, body weight remained stable throughout the study (time, *P* = 0.990; group *P* = 0.170; time-by-group interaction, *P* = 0.988), as shown by weekly averages of daily measurements (Figure 2E). Compared with the CON group, the absolute weight change from baseline to week 12 in the PMR group was 0.58 (−0.19, 1.34) kg (*P* = 0.136), whereas the percentage change was −0.34 (−1.28, 0.61) % (*P* = 0.483). Importantly, only *n* = 2 participants received nutritional counseling from a registered dietitian due to changes >2% of baseline body weight.

### Primary outcome: chronic low-grade inflammation

**FIGURE 3:**
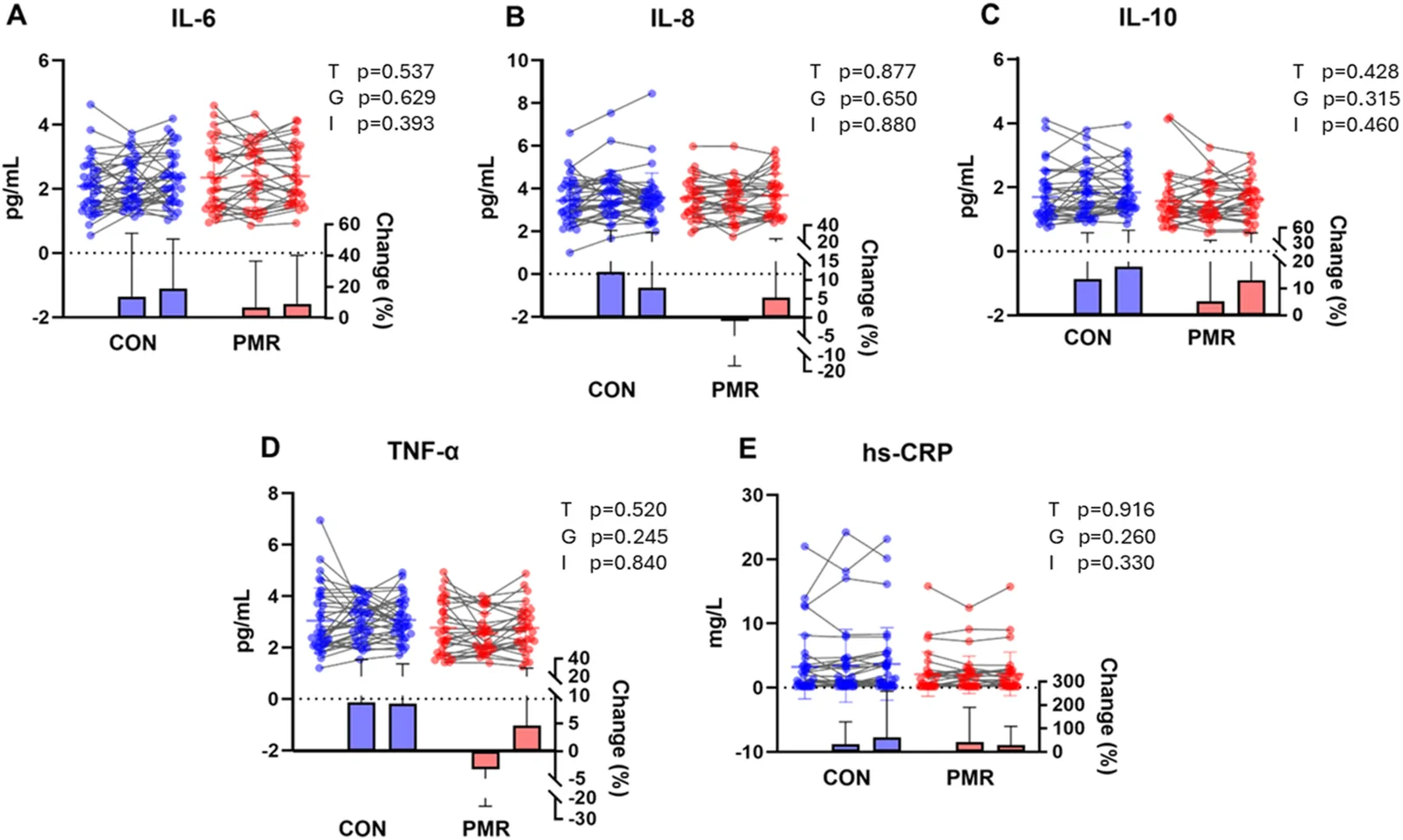
Blood inflammation markers in control (CON, *n* = 34) and powdered meal replacement group (PMR, *n* = 29). (A) IL-6 (picograms per milliliter). (B) IL-8 (picograms per milliliter). (C) IL-10 (picograms per milliliter). (D) TNF-α (picograms per milliliter); (E) High-sensitivity C-reactive protein (hs-CRP, milligrams per liter). Dots represent individual values at baseline, week 6, and week 12, whereas bar graphs below indicate percentage changes from baseline at week 6 and week 12. GEE (all outcomes): False discovery rate-corrected for multiple testing effects included: time (T), group (G), and time-by-group interaction (I). CON, control group; GEE, generalized estimating equation; hs-CRP, high-sensitivity C-reactive protein; IL, interleukin; PMR, powdered meal replacement group.

None of the inflammation markers changed significantly (Figure 3). This includes the primary outcome IL-6, which had a low statistical power [*P*-interaction = 0.393, χ^2^ = 0.729, ES = 0.108, OP = 13.7%; change relative to CON from baseline to week 12 = −0.01 [(−0.47, 0.45) pg/mL, *P* = 0.412]. Likewise, sex-stratified analysis presented no significant effects in any inflammation marker.

### Secondary outcome: gut microbiota

Among study completers, 4 females in the CON group missed 1 of their 3 stool sample collections (2 at week 6 and 2 at week 12). During quality control, 3 samples with low read counts were removed, 2 females from the CON group (1 on week 6 and 1 on week 12) and 1 male from the PMR group (week 6). Participants with missing samples were included in gut microbiota analysis, but missing data were not imputed for a total of *n* = 182 samples (Figure 1).

**FIGURE 4:**
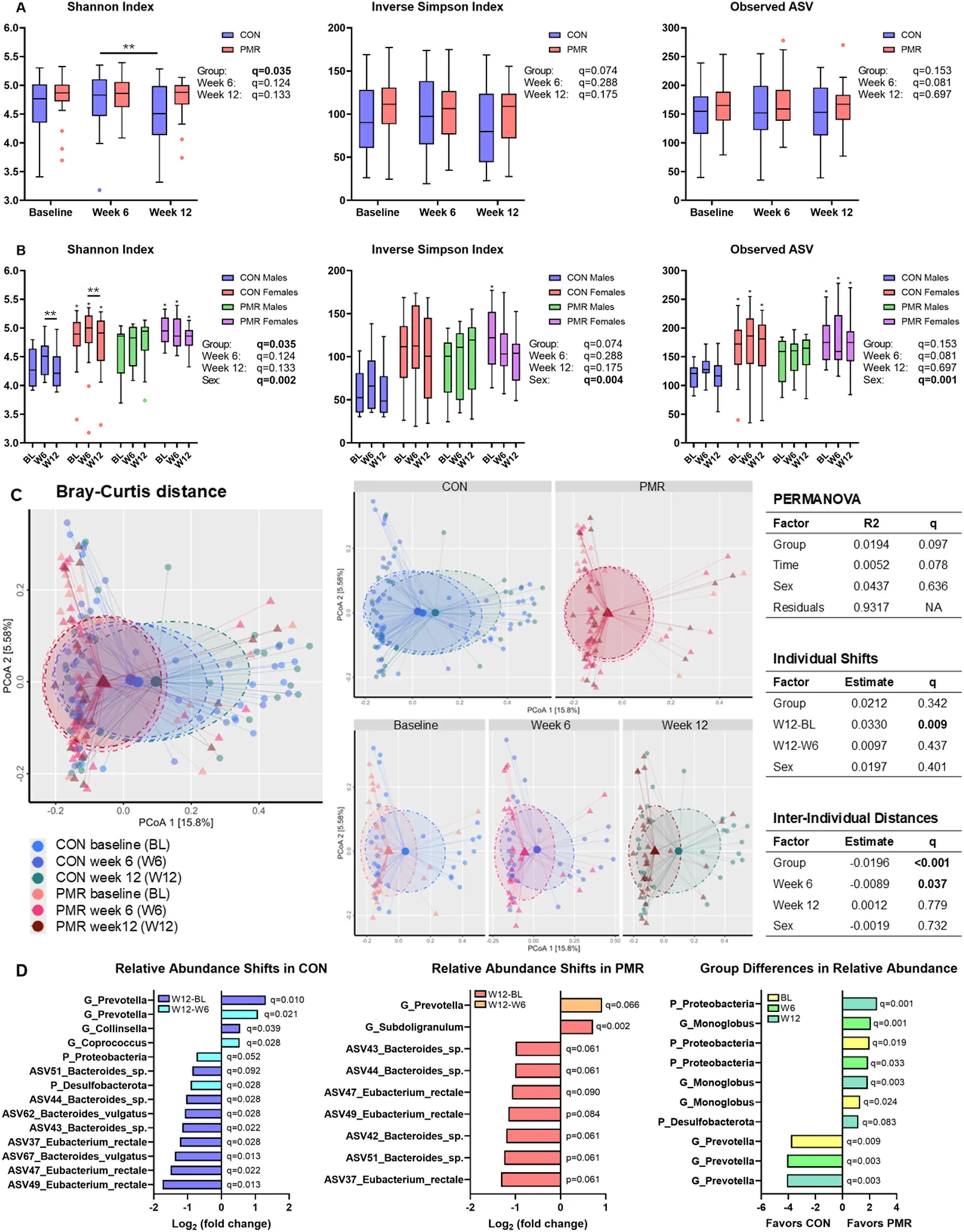
Gut microbiota outcomes in control (CON) and powdered meal replacement (PMR) groups at baseline (BL), week 6 (w6), and week 12 (w12). (A) α-diversity metrics (Shannon index, Inverse Simpson index, and Observed ASV). (B) sex differences in α-diversity metrics. (C) Bray–Curtis β-diversity Principal Coordinate Analysis (PCoA). (D) Waterfall plots representing shifts in relative abundance in each group and differences between groups (Log_2_ fold differences) at the phylum (P), genus (G), and Amplicon Sequence Variant (ASV) levels. All outcomes are False discovery rate-adjusted (*q*), ∗ indicates *q* < 0.1 compared with males, and ∗∗ indicates *q* < 0.05 between time points. The reference categories for linear mixed models were CON group, baseline, and females for α-diversity metrics and β-diversity interindividual distances; and CON group, shift from baseline to week 6, and females for individual shifts of β-diversity. ASV, amplicon sequence variant; BL, baseline; CON, control group; PCoA, principal coordinate analysis; PMR, powdered meal replacement group; w6, week 6; w12, week 12.

Among α-diversity metrics (Figure 4A), the Shannon index differed by group (*q* = 0.035) and decreased from week 6 to week 12 in the CON group (*q* = 0.014). Other α-diversity metrics had no significant effects, though there were trends for a group effect on Inverse Simpson index (*q* = 0.074) and a time effect (week 6 compared with baseline) on observed ASVs (q = 0.081), without significant post hoc differences. Sex differences (Figure 4B) were observed in all α-diversity metrics (Shannon *q* = 0.002, Inverse Simpson *q* = 0.004, and observed ASVs *q* = 0.001). In post hoc analysis, males tended to have lower Shannon index (*q* = 0.085) and observed ASVs (*q* = 0.052) than females in both groups and at each time point. Within the CON group, both males and females reduced the Shannon index from week 6 to week 12 (both *q* = 0.049). There was a trend for sex difference in the Inverse Simpson index only in the PMR group at baseline (*q* = 0.079).

PERMANOVA of Bray–Curtis PCoA distance controlled for repeated measures (Figure 4C) revealed no PMR impact on microbial community structure. Although group allocation explained 1.94% of variation (*q* = 0.097), time explained 0.52% (*q* = 0.078), indicating no changes over time and no group differences. Sex explained 4.37% of the variation but was not significant (*q* = 0.636). A within-individual linear mixed model on Bray–Curtis distances showed that the shift from baseline to week 12 was larger than the shift from baseline to week 6 (*q* = 0.009), without differences between groups, sexes, or between shift from week 6 to week 12. Interindividual distances showed group (*q* < 0.001) and week 6 (*q* = 0.037) effects. Post hoc confirmed group differences at all time points (all *P* < 0.001), but neither group changed over time.

Temporal changes in relative abundance of taxa at phylum, genus, and ASV levels, as well as differences between groups, were minimal (Figure 4D). From baseline to week 12, the CON group had an enrichment in the relative abundance of the genera *Prevotella* (*q* = 0.010) and *Collinsella* (*q* = 0.039), along with reductions in the relative abundance of the species *Eubacterium rectale* (ASV49, *q* = 0.013; ASV47, *q* = 0.022; and ASV37, *q* = 0.028), *Bacteroides vulgatus* (ASV67, *q* = 0.013; ASV62, *q* = 0.028), and *Bacteroides* sp. (ASV43, *q* = 0.022, ASV44, *q* = 0.028). From baseline to week 12, the PMR group showed an enrichment in the relative abundance of the genus *Subdoligranulum* (0.72 Log_2_ fold-change, *q* = 0.002). Group differences in composition were consistent across time points. *Monoglobus* and Proteobacteria were more abundant in PMR, whereas *Prevotella* was more abundant in CON (Figure 4D). At baseline, *Prevotella* was detected in 35% of participants and was more prevalent in CON (*n* = 16, 47.1%) than in PMR (*n* = 6, 20.7%, Fisher’s exact test *P* = 0.036). Additionally, CON had a higher relative abundance of several ASVs of *Prevotella* sp. in all timepoints. The variability in composition within and between groups, including the prevalence of *Prevotella* sp. and its higher-order taxa, is illustrated in Supplementary Figure 1, which depicts individual taxonomic profiles (phylum through species).

### Body composition and energy metabolism

**FIGURE 5:**
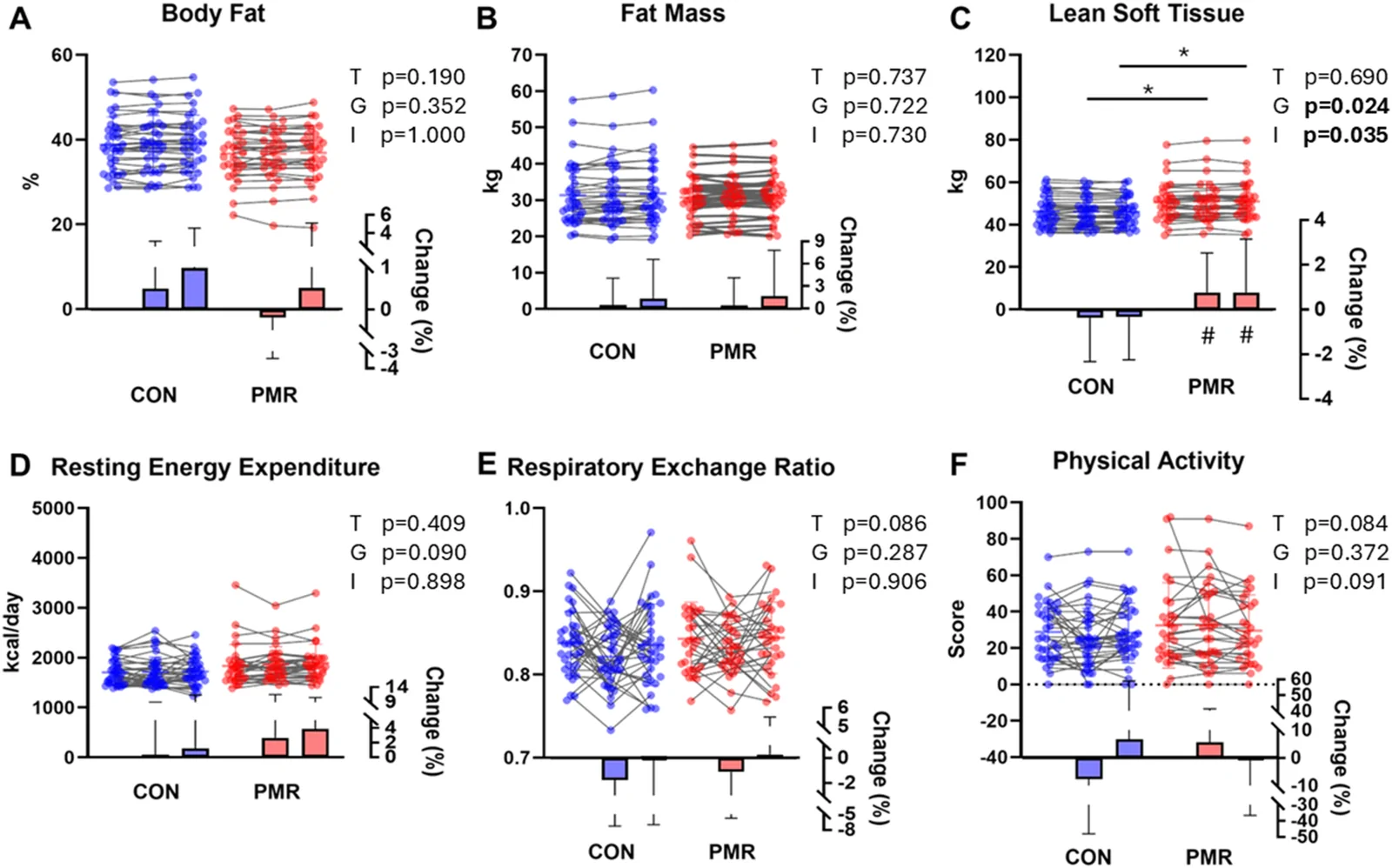
Body composition and energy metabolism-related outcomes in control (CON, *n* = 34) and powdered meal replacement group (PMR, *n* = 29). (A) Body fat (percentage, RM-ANOVA). (B) Fat mass (kilograms, GEE). (C) Lean soft tissue (kilogram, RM-ANOVA). (D) Resting energy expenditure (kilocalories per day, GEE). (E) Respiratory exchange ratio (RM-ANOVA). (F) Physical activity (score, GEE). The dots represent individual values at baseline, week 6, and week 12, whereas bar graphs below indicate percentage changes from baseline at week 6 and week 12. False discovery rate-corrected for multiple testing effects included: time (T), group (G), and time-by-group interaction (I). Group and timepoint differences are indicated by ∗*P* <0.05, whereas between-group differences in changes from baseline to week 6 and week 12 are indicated by ^#^*P* < 0.05 (refer to the text for exact *P* values). CON, control group; GEE, generalized estimating equation; PMR, powdered meal replacement group; RM-ANOVA, repeated measures analysis of variance.

In terms of body composition (Figure 5A–C), LST showed a significant group effect [4.741 (0.638, 8.845) kg, *P* = 0.024, η^2^ = 0.080, ES = 0.295, OP = 62%] and time-by-group interaction (*P* = 0.035, η^2^ = 0.053, ES = 0.237, OP = 63.5%). Relative to the CON group, the PMR group presented increases in LST from baseline to week 6 [0.57 (0.12, 1.02) kg, *P* = 0.014] and from baseline to week 12 [0.57 (0.05, 1.09) kg, *P* = 0.032]. No significant effects were observed for BF%, FM, and bone mineral content (time *P* = 0.324, group *P* = 0.157, interaction *P* = 0.297). There were no significant effects on energy metabolism parameters (REE and RER) after FDR correction, and in physical activity score (Figure 5D–F).

There were expected sex effects on body weight, FM, and LST (all *P* < 0.001). Body weight also showed a significant time effect [*P* = 0.020, week 6 to baseline = 0.40 (0.14, 0.78) kg, *P* = 0.040] and FM had a significant FDR-corrected time-by-sex effect on FM (*P* = 0.036) but with no between-sex differences in changes from baseline. LST showed significant group [3.76 (0.71, 6.81), *P* = 0.017], time-by-group (*P* = 0.005), and time-by-group-by-sex effects (*P* = 0.021). Relative to the CON group, males in the PMR group gained LST from baseline to week 6 [1.19 (0.55, 1.84) kg, *P* = 0.001] and from baseline to week 12 [1.53 (0.65, 2.41) kg, *P* = 0.002], whereas no between-group differences in changes were observed in females (Supplementary Figure 2A). There were no significant effects on BF%, physical activity, and energy metabolism parameters, aside from the expected sex effects on BF% and REE (both *P* < 0.001).

### Blood metabolic markers

**FIGURE 6:**
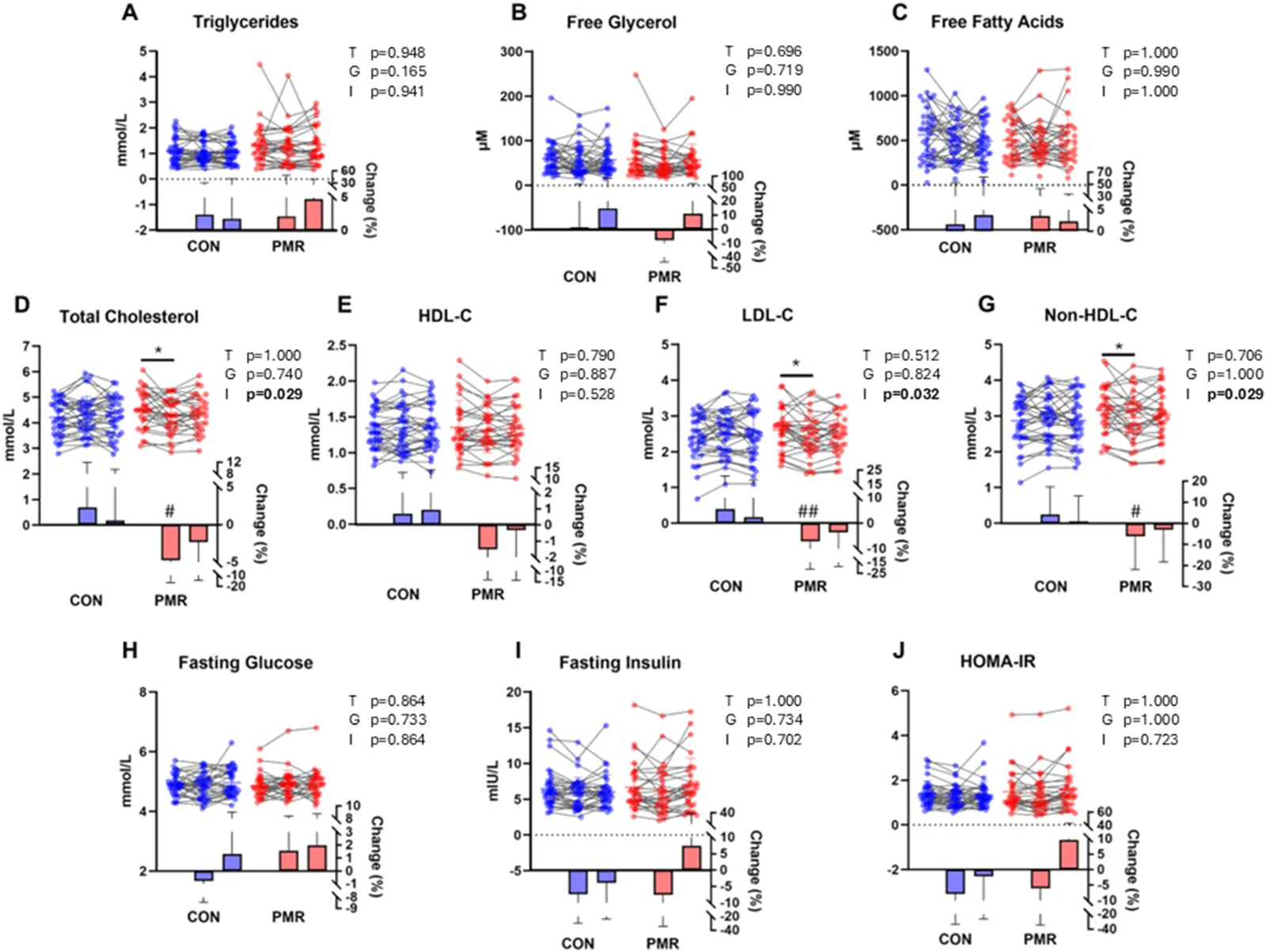
Blood metabolic markers in control (CON, *n* = 34) and powdered meal replacement group (PMR, *n* = 29). (A) Triglycerides (mmol/L, GEE). (B) Free glycerol (μM, GEE). (C) Free fatty acids (μM, GEE). (D) Total cholesterol (mmol/L, RM-ANOVA). (E) HDL cholesterol (mmol/L, RM-ANOVA). (F) LDL cholesterol (mmol/L, RM-ANOVA). (G) Non-HDL cholesterol (mmol/L, RM-ANOVA). (H) fasting glucose (mmol/L, GEE). (I) fasting insulin (mIU/L, GEE). (J) HOMA-IR (GEE). Dots represent individual values at baseline, week 6, and week 12, whereas bar graphs below indicate percentage changes from baseline at week 6 and week 12. FDR-corrected for multiple testing effects included: time (T), group (G), and time-by-group interaction (I). Differences between timepoints are indicated by ∗*P* < 0.05, whereas between-group differences in changes from baseline to week 6 and week 12 are indicated by ^#^*P* < 0.05 and ^##^*P* < 0.01 (refer to the text for exact *P* values).

Triglycerides, free glycerol, and free fatty acids showed no significant effects (Figure 6A–C). For the cholesterol fractions (Figure 6D–G), significant FDR-corrected time-by-group interactions were observed for total cholesterol (*P* = 0.029, η^2^ = 0.061, ES = 0.255, OP = 70.1%), LDL cholesterol (*P* = 0.032, η^2^ = 0.077, ES = 0.289, OP = 81.2%), and non-HDL cholesterol (*P* = 0.038, η^2^ = 0.063, ES = 0.259, OP = 71.7%). Compared with the CON group, the PMR group presented reductions in these markers from baseline to week 6 [total cholesterol = −0.33 (−0.58, −0.08) mmol/L, *P* = 0.001; LDL cholesterol = −0.28 (−0.47, −0.10) mmol/L, *P* = 0.003; and non-HDL cholesterol = −0.29 (−0.51, −0.07) mmol/L, *P* = 0.010]. Fasting glucose, insulin, and HOMA-IR did not change in either group (Figure 6H–J). Hormones related to metabolism and appetite also showed no significant effects (Supplementary Figure 3). Likewise, there were no changes over time in subjective appetite sensation in the PMR group (Supplementary Figure 3).

In sex differences subgroup analysis, FDR-corrected time-by-group effects remained significant for total cholesterol (*P* = 0.047), LDL (*P* = 0.044), and non-HDL cholesterol (*P* = 0.034) (Supplementary Figure 2B). From baseline to week 6, females in the PMR group had significant reductions in LDL cholesterol [−0.30 (−0.53, −0.08), *P* = 0.009] relative to the CON group. From baseline to week 12, males in PMR group had reductions in LDL cholesterol [−0.37 (−0.67, −0.07), *P* = 0.019] and non-HDL cholesterol [−0.45 (−0.79, −0.10), *P* = 0.015]. There were no significant effects on the remaining blood markers.

### Correlations between clinical parameters

Strong positive correlations (corrected for multiple testing) between REE and LST were present at all timepoints (baseline rho = 0.792, week 6 rho = 0.817, week 12 rho = 0.781, all *P* < 0.001). Negative weak to moderate correlations between REE and BF% were also observed in all timepoints (baseline rho = −0.393, *P* = 0.039; week 6 rho = −0.435, *P* = 0.010; week 12 rho = −0.439, *P* = 0.009). No correlations between body composition compartments and RER were observed.

**FIGURE 7:**
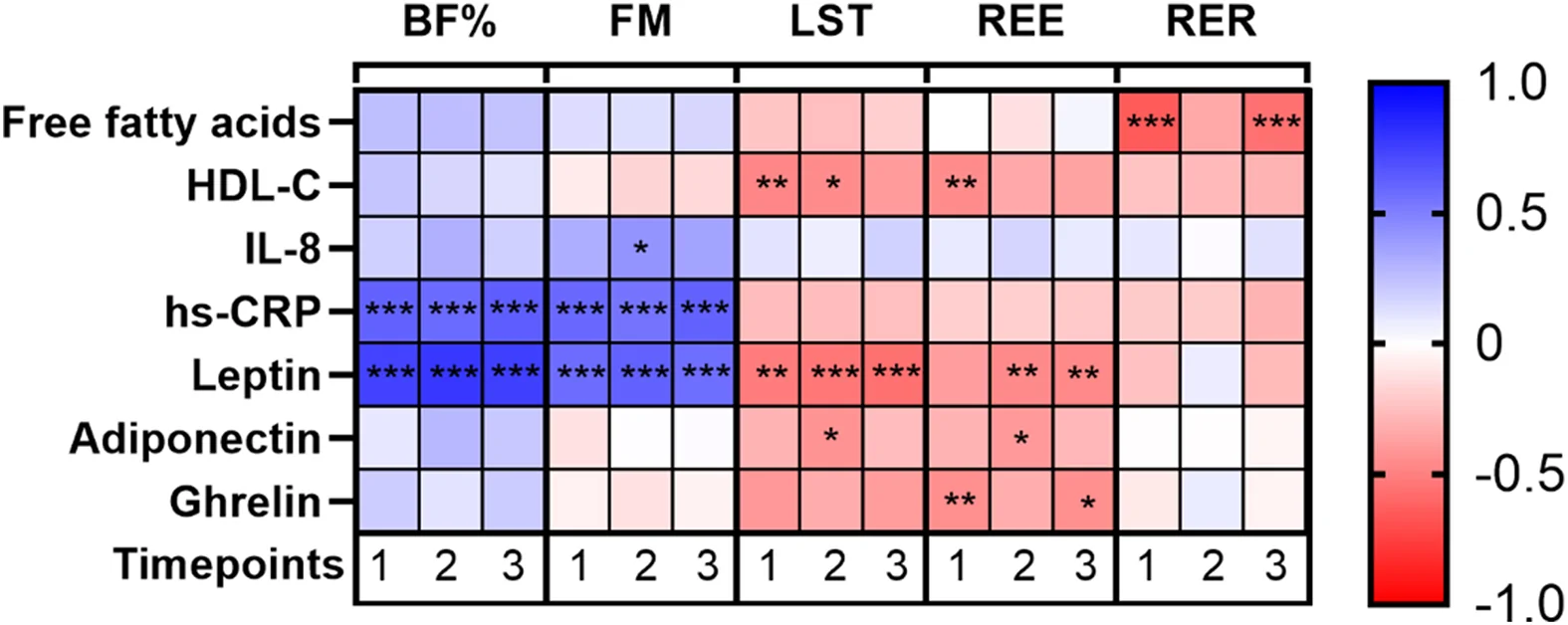
Correlations between blood markers with body composition and energy metabolism FDR-corrected for multiple testing. Outcomes were assessed at the time points: (1) baseline, (2) week 6, and (3) week 12. Significant Bonferroni-corrected correlations are indicated as ∗*P* < 0.05, ∗∗*P* < 0.01, and ∗∗∗*P* < 0.001, refer to the text for exact Spearman’s correlation coefficients (rho) and p values. BF%, body fat percentage; FM, fat mass; hs-CRP, high-sensitivity C-reactive protein; LST, lean soft tissue; REE, resting energy expenditure; RER, respiratory exchange ratio.

We examined how blood markers related to body composition and energy metabolism (Figure 7). Free fatty acids had moderate negative correlations with RER (baseline rho = −0.646, week 12 rho = −0.563, both *P* < 0.001). Among inflammation markers, hs-CRP had moderate to strong correlations with BF% (baseline rho = 0.615; week 6 rho = 0.582; and week 12 rho = 0.633; all *P* < 0.001) and FM (baseline rho = 0.590; week 6 rho = 0.544; and week 12 rho = 0.615; all *P* < 0.001). Leptin had strong correlations (all *P* ≤ 0.001) with BF% (baseline rho = 0.738; week 6 rho = 0.784; and week 12 rho = 0.751), moderate with FM (baseline rho = 0.574; week 6 rho = 0.618; and week 12 rho = 0.567) and LST (baseline rho = −0.515; week 6 rho = 0.534; and week 12 rho = 0.561). Leptin also had weak to moderate correlations with REE (week 6 rho = 0.462, *P* = 0.005; and week 12 rho = 0.467, *P* = 0.004).

We performed correlation tests between inflammation markers and other blood markers, between blood markers and dietary intake, and between appetite-regulating hormones and subjective appetite sensation. After correction for multiple testing, all those correlations were insignificant (data not shown). Additionally, we performed correlations between gut microbiota and clinical outcomes, including controlled by group assignment; these were insignificant after FDR correction (data not shown).

## Discussion

This study assessed whether PMR could improve chronic low-grade inflammation, gut microbiota, and metabolism in people with excess body weight without weight loss. Overall, adding PMR to a habitual diet did not change inflammation markers, including the primary outcome IL-6, or alter gut microbiota composition, the secondary outcome. Instead, PMR intake improved lipid profiles and increased LST, highlighting direct benefits beyond the indirect cardiometabolic effects seen with weight loss [[18], [19], [20], [21], [22], [23], [24], [25], [26], [27], [28]]. This finding is particularly relevant because achieving and maintaining weight loss remains challenging for many individuals [61]. Although it cannot replace a healthy lifestyle, PMR can support dietary and exercise efforts to manage excess body weight and obesity comorbidities.

The addition of PMR to the usual diet of individuals with excess body weight led to transient improvements in cholesterol fractions even in the absence of weight loss and despite most participants not presenting dyslipidemia at baseline. These findings suggest that the total cholesterol and LDL cholesterol lowering effects of PMR may be further amplified in individuals with dyslipidemia. This is clinically meaningful because dyslipidemia is a major risk factor for various cardiovascular diseases, in part due to the atherogenic role of LDL cholesterol, which contributes to cholesterol plaque formation in blood vessels [62]. In pharmacological RCTs, each 1 mmol/L reduction in LDL cholesterol is associated with a 12% lower risk of cardiovascular events within 1 y [63]. In the current study, LDL cholesterol reductions were smaller, consistent with what is expected in nutritional interventions (∼5%), yet such changes may still have clinically relevant effects, particularly when combined with other behavioral interventions [64]. Findings from the present study align with previous RCTs reporting improvements in lipid profile among participants consuming PMR compared with controls [25,26].

Previous RCTs in individuals with excessive body weight have shown that PMR combined with weight loss improved glucose control markers (fasting glucose, fasting insulin, HOMA-IR, and glycated hemoglobin), IL-6, hs-CRP, and leptin more effectively than control lifestyle interventions [18,[20], [21], [22], [23], [24], [25], [26], [27], [28]], indicating that weight loss may be necessary for meaningful changes in those markers, as previously reported [65]. Regarding glucose control markers, some of those studies included patients with diabetes or prediabetes [[23], [24], [25]], whereas in our current study, those markers were within normal range. Some participants had elevated hs-CRP and leptin concentrations in the present study, but the considerable heterogeneity in those markers likely limited statistical power to detect changes.

Despite weight stability, LST increased among participants in PMR, particularly in males. Previous trials showed PMR consumption preserved fat-free mass and LST during weight loss [66,67]. These findings indicate that PMR supports muscle maintenance during weight loss and promotes muscle gain during weight stability. This likely reflects the increased protein intake, which supports skeletal muscle mass maintenance and growth, especially when combined with resistance exercise [68]. There is no established threshold for clinically significant increase in LST. Although the increase observed here was modest (∼600 g in the combined sample, and ∼1.5 kg in males relative to the CON group), even small gains or maintenance can be clinically meaningful depending on context (baseline muscle status, sex, age, and disease). Also, it may be particularly important in individuals who are at risk of losing muscle mass and strength (i.e., sarcopenia). Although sarcopenia is most commonly age-related and not applicable to the present study population, it can also develop secondarily from other conditions, including obesity (sarcopenic obesity), due to factors such as increased oxidative stress, insulin resistance, chronic low-grade inflammation, and fat infiltration into muscle [69,70]. Additionally, the LST increase was observed without exercise intervention, combining PMR with exercise could yield greater gains. This increase is also promising for other scenarios, such as during aging or weight loss with GLP-1 receptor agonists [71].

For gut microbiota composition, both groups exhibited similar shifts over time. The CON group had additional taxonomic changes and reductions in Shannon α-diversity despite no clinical or dietary alterations that would explain variations, indicating random fluctuation. Consequently, trends observed in the PMR group cannot be attributed to PMR intake. Notably, the only taxon to increase significantly only in PMR was *Subdoligranulum*, suggesting possible enrichment by the intervention. *Subdoligranulum* has been associated with health benefits, including butyrate production, inverse correlation with FM, insulin HOMA-IR, leptin, IL-6, and hs-CRP, and positive correlation with HDL cholesterol and adiponectin [72]. However, there is a lack of causational evidence for *Subdoligranulum* improving metabolic outcomes [72]. Moreover, although statistically significant, a change in the relative abundance of a single genus does not indicate a relevant alteration in the microbial community structure or function [73], consistent with the absence of changes in α- and β-diversity over time. Group differences are probably driven by background diet rather than intervention. Taxonomic analysis confirmed that PMR participants consistently had a lower relative abundance of the genus *Prevotella* and its ASVs compared with CON. Since *Prevotella* prevalence in developed countries is generally low (genus, ∼25%–50%; individual species, ∼0%–20%) [74], group differences in relative abundance of these taxa reflect greater random assignment of *Prevotella*-containing participants to the CON group, confirmed by differences in prevalence. The overall stability of the PMR microbiota and lack of significant correlations imply that clinical improvements were not driven by major compositional shifts, although *Subdoligranulum* merits further investigation. Future work should also assess functional and metabolic changes to clarify any microbiota-related mechanisms underlying any clinical improvements, particularly in lipid profiles.

This study has several strengths, including the RCT design, which is the reference standard for clinical studies because it minimizes selection bias. This study also benefited from comprehensive assessments, allowing a thorough appraisal of the impact of PMR on metabolism. In addition, the use of advanced technologies, such as WRIC, DXA, and 16S rRNA sequencing, ensured accurate and precise assessments and robustness of findings. Moreover, by maintaining weight stability, the study isolated the effects of PMR without the confounding effect of weight loss. However, this study also has some limitations, mainly, it could not be double-blind, as no inert placebo that resembles the PMR formula is available. Physical activity levels and PMR adherence were self-reported. Future studies could use accelerometers to track physical activity to reduce reporting bias [75]. Intention-to-treat analysis was not feasible as some participants were withdrawn after initiating medications that rendered them ineligible. Consequently, the completer-only analysis was conducted, which could have introduced attrition bias, potentially influencing the estimated intervention effects and limiting the generalizability of the findings. Quantification of soy isoflavone metabolites in urine and blood is planned as a biomarker of adherence to PMR intake [76,77], allowing a biomarker-based per-protocol analysis. Additionally, the dropout rate was higher than anticipated, which may have increased the risk of type II error. However, recruitment followed pre-specified protocol and was not extended beyond the planned enrollment target. Given the higher than anticipated accrual, particularly in the PMR group, we might not have achieved statistical power. Indeed, we observed a low power of 14% for the primary outcome, IL-6, (χ^2^ = 0.729, ES = 0.108), which reflects a limitation in the study design and sample size calculation. However, we observed moderate power for other clinical outcomes, reaching up to 81.2% for LDL cholesterol (η^2^ = 0.077, ES = 0.289). Another limitation is the relatively short intervention duration (12 weeks), especially when compared with the study used for sample size calculation (12 months) [48]. The addition of PMR twice daily may also not be sustainable in a real-world scenario over the long term, as adherence may be lower due to taste fatigue and costs [78,79]. Conversely, PMR adherence may be higher than with regular food-based interventions as it is easier to incorporate into daily routine [78]. The higher proportion of females than males and individuals with overweight than those with obesity also limit the generalizability of findings. In addition, the heterogeneity observed in certain outcomes reduces the statistical power limiting the ability to detect significant results. Future studies should consider screening participants for elevated inflammation markers to reduce heterogeneity and evaluate whether PMR improves inflammation specifically in individuals with confirmed chronic low-grade inflammation. An exploratory secondary analysis creating a score for inflammation and other parameters could also be performed to minimize false negative findings.

In conclusion, the addition of PMR to the habitual diet underweight-stable conditions in individuals with excess body weight did not change markers of chronic low-grade inflammation, including the primary outcome IL-6. Only minimal changes in gut microbiota composition, the secondary outcome, were observed. However, PMR intake was associated with modest improvements in lipid profiles and an increase in LST, both exploratory outcomes.

## Author contributions

The authors’ responsibilities were as follows – CMP, JW: aquired funding for the study; JM, CLPO, NKN, AMA, AB, AMS, CC, SG, PDC, CR, JW, CMP: designed the study; JM, CLPO, AMS, LM: participated in recruitment; JM, CLPO: collected data; JM, NKN, SG: analyzed data; JM, CLPO, AMA, AB, CR, JW, CMP: performed data interpretation; JM: drafted the manuscript; CMP, JW: provided study supervision and management; and all authors revised, edited, and approved the final manuscript.

## Data availability

Data described in the manuscript will be made available upon request.

## Declaration of Generative AI and AI-Assisted Technologies in the Writing Process

The author(s) declare that no generative AI or AI-assisted technologies were used in the writing of this manuscript.

## Funding

This was an investigator-initiated trial supported by Almased Wellness-GmbH (München, Germany). Per contractual agreement, the funder had no role in the study design and implementation, results interpretation, writing of the manuscript, and decision to submit the article for publication. Some of the infrastructure used in the project was funded by the Canadian Foundation for Innovation John R Evans Leaders Fund (Project # 34115). CMP was partially funded by the Canada Research Chair and Campus Alberta Innovates Programs. The Canada Research Chair Program partially funded CR. CLPO was supported by the Mitacs Accelerate International (Mitacs, Canada) in partnership with Almased USA Inc. PDC is an honorary research director at Fonds de la Recherche Scientifique (FNRS) and is a recipient of grants from FNRS, which supported research staff (Projet de Recherche PDR-convention: FNRS T.0032.25, CDR-convention: J.0027.22, FRFS-WELBIO: WELBIO-CR-2022A-02P, EOS: program no. 40007505).

## Conflict of interest

In addition to what is noted under “Funding,” JM was supported by the Alberta Diabetes Institute and has received a traveling grant from Fresenius Kabi. CLPO reports receiving honoraria and/or paid consultancy from Abbott Nutrition and AMRA Medical Inc. outside the scope of this work. AB received research support for his department and consultant/speakers’ honoraria from the Almased Wellness-GmbH. AMS reports receiving honoraria and/or paid consultancy from AbbVie, Amgen, AstraZeneca, Currax, Lilly, Novo Nordisk, and Boehringer Ingelheim outside the scope of this work. PDC is an inventor on patent applications dealing with the use of bacteria in metabolic disorders, and a co-founder of Enterosys, outside the scope of this work. JW has received research funding and consulting fees from industry sources involved in the manufacture and marketing of dietary fibers, prebiotics, and probiotics. JW is also a co-owner of Synbiotics Health, a developer of synbiotic products, outside the scope of this work. CMP reports receiving honoraria and/or paid consultancy from Abbott Nutrition, Nutricia, Nestle Health Science, and Novo Nordisk, outside the scope of this work. Other authors declare no conflict of interest.
