# Supplementary material for: The Effects of a Powdered Meal Replacement on Inflammation, Gut Microbiota, and Metabolism in People With Excess Body Weight—A Randomized Controlled Trial
Source: J Nutr. 2026 May 15;156(7):101595. doi: 10.1016/j.tjnut.2026.101595 (PMC13494188; doi:10.1016/j.tjnut.2026.101595)
Supplement: Supplementary file 1 [file mmc1.docx]

**The effects of a powdered meal replacement intake on inflammation, gut microbiota, and metabolism compared to habitual diet in people with excess body weight – results from a randomized controlled trial**

Julia Montenegro, Camila L. P. Oliveira, Nguyen Khoi Nguyen, Anissa M. Armet, Aloys Berg, Arya M. Sharma, Laurie Mereu, Cristiane Cominetti, Sunita Ghosh, Patrice D Cani, Caroline Richard, Jens Walter, Carla M. Prado

**Supplementary Table 1.** Nutritional information for the investigational product (Almased Original, Almased Wellness-GmbH, München, Germany).

| **Nutrient** | **Per dose (50 g)** | **Per 100 g** |
| --- | --- | --- |
| Calories (kcal) | 180 | 360 |
| Total fat (g) | 1.0 | 2.0 |
| *Saturated fat (g)* | 0.5 | 1.0 |
| *Trans fat (g)* | 0 | 0 |
| *Polyunsaturated fat (g)* | 0.1 | 0.2 |
| *Monounsaturated fat (g)* | 0.4 | 0.8 |
| Cholesterol (mg) | 3 | 6 |
| Total carbohydrates (g) | 15 | 30 |
| *Dietary fiber (g)* | 0.5 | 1.0 |
| *Sugars (g)* | 15 | 30 |
| Protein (g) | 27 | 54 |
| Sodium (mg) | 340 | 680 |
| Potassium (mg) | 500 | 1000 |
| Vitamin A (IU) | 794 | 1588 |
| Vitamin C (mg) | 16 | 32 |
| Vitamin E (IU) | 6 | 12 |
| Thiamin (Vitamin B1) (mg) | 5 | 10 |
| Riboflavin (Vitamin B2) (mg) | 6 | 12 |
| Vitamin B6 (mg) | 7 | 14 |
| Calcium (mg) | 215 | 430 |
| Iron (mg) | 4.9 | 9.8 |

**Supplementary Table 2.** Baseline characteristics comparisons between participants who completed or dropped out of the study in the control (CON) and powdered meal replacement (PMR) arms.

| Variable | Completers | | Dropouts | | P ^a^ |
| --- | --- | --- | --- | --- | --- |
|  | CON (n=34) | PMR (n=29) | CON (n=3) | PMR (n=9) ^b^ |  |
| Sex (females:males) | 24:10 | 18:11 | 1:2 | 7:2 | - |
| Age (years) | 28 (23, 33) | 28 (23, 35) | 29 (25, 30) | 27 (24, 29) | 0.434 |
| Height (m) | 1.67 ± 0.09 | 1.71 ± 0.10 | 1.72 ± 0.04 | 1.65 ± 0.12 | 0.584 |
| Weight (kg) | 80.2 (72.4, 86.7) | 83.2 (79.3, 88.7) | 80.6 (77.6, 84.5) | 76.7 (71.3, 90.3) | 0.618 |
| BMI (kg/m^2^) | 28.1 (27.3, 29.8) | 29.1 (26.6, 30.4) | 25.9 (25.6, 28.5) | 29.3 (28.7, 31.9) | 0.784 |
| Body fat (%) | 38.76 ± 6.57 | 36.67 ± 6.08 | 32.03 ± 0.95 | 42.03 ± 3.44* | 0.568 |
| Fat mass (kg) | 28.86 (25.16, 36.30) | 30.19 (27.71, 33.28) | 24.7 (24.6, 26.3) | 31.4 (29.3, 39.5) | 0.797 |
| LST (kg) | 46.24 ± 7.02 | 50.60 ± 9.29 | 51.84 ± 4.49 | 44.73 ± 11.36 | 0.637 |
| REE (kcal/day) | 1662 (1488, 1863) | 1748 (1561, 1909) | 1819 (1650, 1825) | 1610 (1519, 1928) | 0.723 |
| RER | 0.837 ± 0.038 | 0.843 ± 0.044 | 0.885 ± 0.038* | 0.829 ± 0.058 | 0.813 |
| Triglycerides (mmol/L) | 1.05 (0.74, 1.37) | 1.23 (0.82, 1.64) | 1.12 (0.95, 1.27) | 1.21 (1.00, 1.80) | 0.394 |
| Free glycerol (μM) | 56.5 (35.8, 76.0) | 45.7 (32.0, 75.2) | 35.5 (32.8, 57.7) | 71.1 (51.7, 85.6) | 0.305 |
| Free fatty acids (μM) | 600.1 (315.9, 771.5) | 492.8 (306.6, 730.7) | 280.2 (250.7, 351.2) | 558.1 (420.9­­, 863.9) | 0.840 |
| Total Cholesterol (mmol/L) | 4.21 ± 0.69 | 4.52 ± 0.75 | 3.92 ± 0.50 | 4.75 ± 0.71 | 0.415 |
| HDL-C (mmol/L) | 1.34 ± 0.29 | 1.35 ± 0.37 | 1.19 ± 0.10 | 1.22 ± 0.20 | 0.166 |
| LDL-C (mmol/L) | 2.40 ± 0.61 | 2.62 ± 0.58 | 2.26 ± 0.54 | 2.91 ± 0.61 | 0.208 |
| Non-HDL-C (mmol/L) | 2.87 ± 0.66 | 3.17 ± 0.66 | 2.73 ± 0.58 | 3.53 ± 0.65 | 0.134 |
| Glucose (mmol/L) | 4.9 (4.6, 5.1) | 4.8 (4.6, 5.1) | 4.8 (4.7, 5.0) | 4.5 (4.4, 5.0) | 0.198 |
| Insulin (mIU/L) | 5.94 (4.62, 7.21) | 5.76 (4.67, 8.34) | 4.93 (4.18, 6.75) | 8.15 (8.03, 9.30) | 0.198 |
| HOMA-IR | 1.29 (1.00, 1.68) | 1.18 (0.98, 1.77) | 1.14 (0.94, 1.46) | 1.63 (1.59, 2.03) | 0.305 |
| IL-6 (pg/mL) | 2.07 (1.41, 2.59) | 2.00 (1.42, 3.13) | 1.73 (1.61, 1.82) | 2.11 (2.03, 2.59) | 0.874 |
| IL-8 (pg/mL) | 3.35 (2.78, 4.18) | 3.51 (2.88, 4.02) | 3.20 (2.97, 3.61) | 4.01 (2.69, 4.36) | 0.675 |
| IL-10 (pg/mL) | 1.40 (1.11, 2.06) | 1.34 (1.01, 1.79) | 1.55 (1.49, 1.64) | 1.48 (1.39, 1.58) | 0.298 |
| TNF-α (pg/mL) | 2.56 (2.12, 3.99) | 2.56 (1.80, 3.73) | 2.79 (2.78, 2.88) | 3.04 (2.51, 4.12) | 0.165 |
| hs-CRP (mg/L) | 1.0 (0.2, 3.4) | 0.6 (0.2, 2.2) | 0.2 (0.2, 0.7) | 1.4 (0.2, 5.2) | 0.988 |
| TSH (mIU/L) | 1.83 (1.24, 2.29) | 1.71 (1.16, 2.53) | 1.92 (1.39, 2.14) | 1.60 (1.22, 2.36) | 0.767 |
| Leptin (ηg/mL) | 6.62 (4.13, 12.14) | 6.10 (4.14, 8.70) | 5.30 (3.65, 7.98) | 8.26 (8.20, 10.35) * | 0.219 |
| Adiponectin (mg/mL) | 4.72 (3.67, 6.31) | 4.62 (3.49, 6.85) | 2.98 (2.68, 5.52) | 5.32 (4.66, 7.42) | 0.583 |
| Active ghrelin (pg/mL) | 240.2 (188.9, 366.8) | 224.8 (184.3, 288.5) | 266.0 (227.8, 359.1) | 222.7 (194.8, 266.4) | 1.000 |
| Active GLP-1 (pMol/L) | 1.05 (0.62, 1.48) | 0.85 (0.62, 1.75) | 1.58 (1.32, 1.62) | 1.42 (1.27, 1.96) | **0.021** |
| PYY (pg/mL) | 118.3 (96.5, 158.4) | 141.4 (108.7, 194.5) | 162.4 (140.4, 179.8) | 140.5 (126.2, 180.5) | 0.254 |

Data expressed as mean ± standard deviation if normally distributed or as median (interquartile interval) if non-normally distributed. Comparisons between subjects who completed the study versus dropouts are provided for descriptive purposes only. Given the small number of dropouts, these analyses are unpowered to detect meaningful differences and should be interpreted cautiously.

^a^ Comparison between completers and dropouts with no distinction between groups (CON or PMR), independent t-test if normally distributed and Mann-Whitney U test if non-normally distributed; * indicate significant difference between completers and dropouts in each allocated group (p<0.05), refer to the text for exact p values. Comparisons did not include correction for multiple testing.

^b^ Body composition data not available for two dropout participants in the PMR group (one male, one female).

Abbreviations: BMI, body mass index; GLP-1, glucagon-like peptide-1; HDL-C, high-density lipoprotein cholesterol; HOMA-IR, Homeostatic Model Assessment for Insulin Resistance; hs-CRP, high-sensitivity C-reactive protein; IL, interleukin; LDL-C, low-density lipoprotein cholesterol; LST lean soft tissue; PYY, peptide tyrosine-tyrosine; REE, resting energy expenditure; RER, respiratory exchange ratio; TNF-α, tumor necrosis factor-α; TSH, thyroid stimulating hormone.

**Supplementary Table 3**. Additional protein and amino acids provided by two daily doses 50g of powdered meal replacement (PMR) formula.

|  | **PMR content** | **Combined (n=29)** | **Males (n=11)** | **Females (n=18)** |
| --- | --- | --- | --- | --- |
| **Protein PMR only (g/kg)** | 54 g | 0.64 ± 0.08 | 0.60 ± 0.09 | 0.67 ± 0.06 |
| **Protein PMR + diet (g/kg)** | - | 1.71 ± 0.37 | 1.65 ± 0.35 | 1.74 ± 0.38 |
| **Histidine (mg/kg)** | 1400 mg | 16.69 ± 2.04 | 15.51 ± 2.34 | 17.41 ± 1.49 |
| **Isoleucine (mg/kg)** | 2800 mg | 33.37 ± 4.09 | 31.01 ± 4.67 | 34.82 ± 2.98 |
| **Leucine (mg/kg)** | 4600 mg | 54.83 ± 6.71 | 50.95 ± 4.89 | 57.20 ± 4.89 |
| **Lysine (mg/kg)** | 3100 mg | 36.95 ± 4.52 | 34.34 ± 5.17 | 38.55 ± 3.29 |
| **Methionine + cysteine (mg/kg)** | 1400 mg | 16.69 ± 2.04 | 15.51 ± 2.34 | 17.41 ± 1.49 |
| **Phenylalanine + tyrosine (mg/kg)** | 4500 mg | 53.64 ± 6.57 | 49.84 ± 7.51 | 55.96 ± 4.78 |
| **Threonine (mg/kg)** | 1900 mg | 22.65 ± 2.77 | 21.04 ± 3.17 | 23.63 ± 2.02 |
| **Tryptophan (mg/kg)** | 800 mg | 9.54 ± 1.17 | 8.86 ± 1.33 | 9.95 ± 0.85 |
| **Valine (mg/kg)** | 2800 mg | 33.37 ± 4.09 | 31.01 ± 4.67 | 34.82 ± 2.98 |

**Supplementary Figure 1**. Gut microbiota composition in individuals in control (CON) or powdered meal replacement (PMR) groups at baseline, week 6, and week 12. Expressed as proportions (relative abundance) of each taxonomic level (phylum, class, order, family, genus, and species).

**Supplementary Figure 2.** Sex-stratified between-group differences (as intervention – control) in changes from baseline to week 6 and week 12 of selected outcomes. **A:** lean soft tissue (kg). **B:** Total cholesterol, low-density lipoprotein cholesterol (LDL-C), and non-high-density lipoprotein cholesterol (Non-HDL-C; all in mmol/L). Outcomes were selected based on significant interaction effects assessed by two-way repeated measurements ANOVA after FDR correction for multiple testing. Effects are indicated as group (G), time (T), sex (S) and interactions. Significant group differences regarding changes over time in each sex are indicated by * p<0.05 and ** p<0.001.

**Supplementary Figure 3.** Hormone related to metabolism and appetite in control (CON, n=34) and powdered meal replacement group (PMR, n=29). **A:** Thyroid-stimulating hormone (TSH, mIU/L). **B:** Leptin (ηg/mL). **C:** Adiponectin (mg/mL). **D:** Leptin-to-adiponectin ratio. **E:** Active ghrelin (pg/mL). **F:** Active glucagon-like peptide-1 (GLP-1, pmol/L). **G:** Peptide tyrosine-tyrosine (PYY, pg/mL). Dots represent individual values at baseline, week 6, and week 12, while bar graphs below indicate percentage changes from baseline at week 6 and week 12. GEE (all outcomes) effects included: time (T), group (G), and time-by-group interaction (I). **H:** Weekly subjective appetite sensations (hunger, satisfaction, fullness, and prospective food consumption [PFC]) evaluated only in the PMR group as repeated measures ANOVA of area under curve (AUC) of five measurements in one day (fasting upon waking up, pre-morning PMR, 30 min post-morning PMR, pre-afternoon PMR, and 30 min post-afternoon PMR). All analyses were FDR-corrected for multiple testing.
